# Ipsilateral somatic nerves mediate histamine-induced vasosensory reflex responses involving perivascular afferents in rat models

**DOI:** 10.1038/s41598-021-94110-x

**Published:** 2021-07-19

**Authors:** Ravindran Revand, Sanjeev K. Singh

**Affiliations:** Department of Physiology, Institute of Medical Sciences, Banaras Hindu University, Varanasi, Uttar Pradesh 221005 India

**Keywords:** Physiology, Cardiovascular biology, Neurophysiology

## Abstract

Reflex cardiorespiratory alterations elicited after instillation of nociceptive agents intra-arterially (i.a) are termed as ‘vasosensory reflex responses’. The present study was designed to evaluate such responses produced after i.a. instillation of histamine (1 mM; 10 mM; 100 mM) and to delineate the pathways i.e. the afferents and efferents mediating these responses. Blood pressure, electrocardiogram and respiratory excursions were recorded before and after injecting saline/histamine, in a local segment of femoral artery in urethane anesthetized rats. Paw edema and latencies of responses were also estimated. Separate groups of experiments were conducted to demonstrate the involvement of somatic nerves in mediating histamine-induced responses after ipsilateral femoral and sciatic nerve sectioning (+NX) and lignocaine pre-treatment (+Ligno). In addition, another set of experiments was performed after bilateral vagotomy (+VagX) and the responses after histamine instillation were studied. Histamine produced concentration-dependent hypotensive, bradycardiac, tachypnoeic and hyperventilatory responses of shorter latencies (2–7 s) favouring the neural mechanisms in eliciting the responses. Instillation of saline (time matched control) in a similar fashion produced no response, excluding the possibilities of ischemic/stretch effects. Paw edema was absent in both hind limbs indicating that the histamine did not reach the paws and did not spill out into the systemic circulation. +NX, +VagX, +Ligno attenuated histamine-induced cardiorespiratory responses significantly. These observations conclude that instillation of 10 mM of histamine produces optimal vasosensory reflex responses originating from the local vascular bed; afferents and efferents of which are mostly located in ipsilateral somatic and vagus nerves respectively.

Tissues exposed to nociceptive stimuli undergo damage and repair by mechanisms influenced by several inflammatory mediators. Histamine, serotonin, bradykinin, prostaglandins and substance P are notable among them. Besides playing a key role in the functional restoration of damaged tissues, these chemical mediators themselves demonstrate certain nociceptive properties. These nociceptive agents present in the circulation can stimulate the free nerve endings located in various parts of the body. The reflex cardiorespiratory (CVR) responses evoked by such nociceptive agents, when instilled in blood vessels are termed as ‘vasosensory reflex responses’^1, 2^. The receptors for these reflexes are situated on the perivascular sensory nerve endings of the peripheral blood vessels^2, 3^.These perivascular afferents get activated by chemical mediators and cause reflex CVR changes through mechanisms involving the central nervous system^3^.

The vasosensory reflexes elicited by various nociceptive agonists like capsaicin, anandamide, αβ Me-ATP and bradykinin have been described elsewhere^2–6^.Works from our department have also demonstrated vasosensory reflexes using *Mesobuthus tamulus* (BT) venom and bradykinin as a nociceptive tool^1, 7–10^. Histamine is a principal mediator of inflammation and a pure nociceptive agonist, but the nature of vasosensory reflex responses elicited after intra-arterial (i.a.) injection of histamine has not yet been described so far, to the best of our knowledge. Further, we have developed a novel experimental design in which the minimum and constant volume of nociceptive agonist can be deposited in a segment of an artery^11^. In the present study, histamine was used as a tool for the elicitation of vasosensory reflex responses. Therefore, this study was undertaken to demonstrate the effects of i.a instillation of histamine in a segment of an artery and also the role of ipsilateral somatic and vagus nerves in producing the responses (Fig. 1).

**Figure 1:**
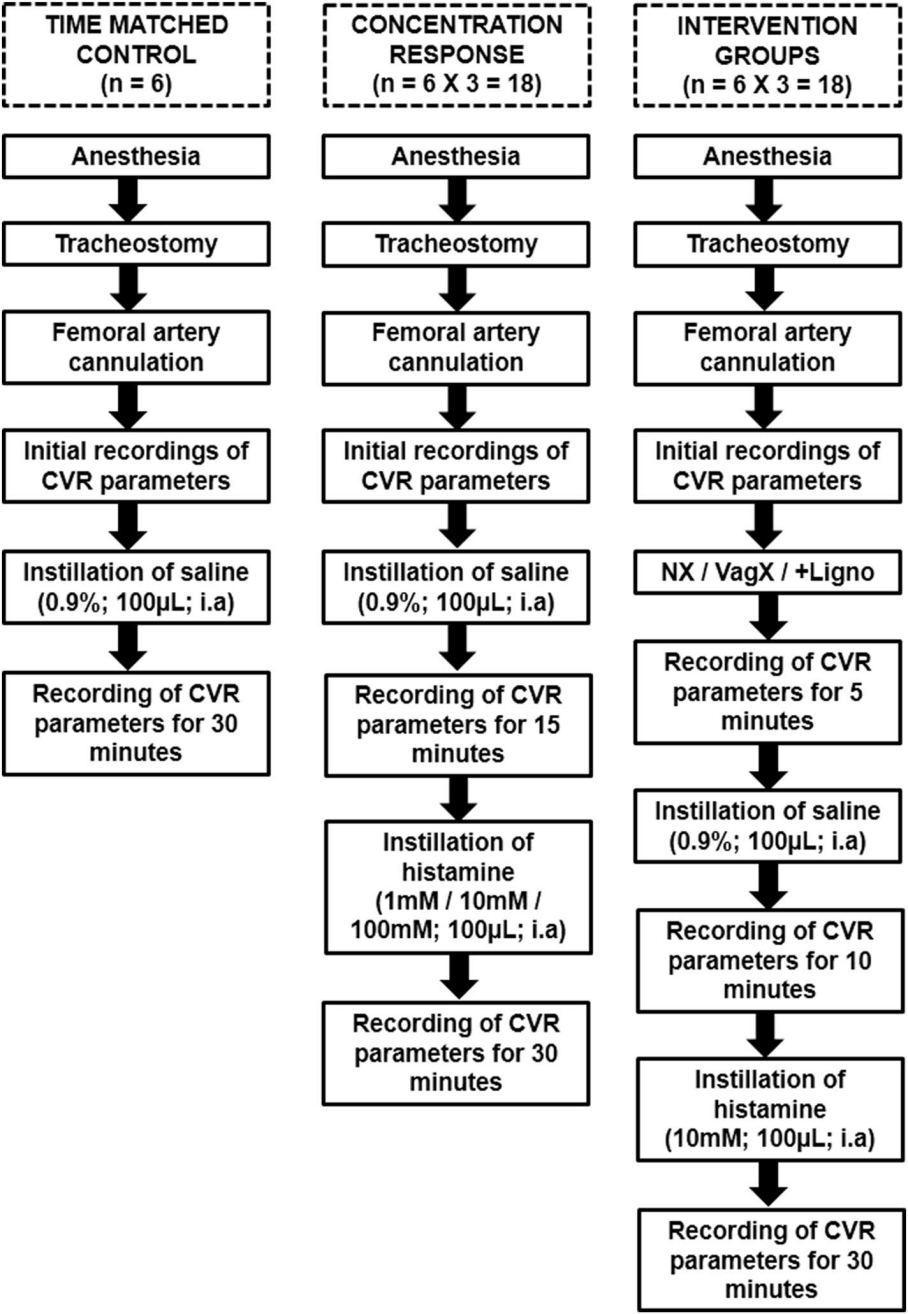
Flowcharts illustrating the experimental protocol. In the first part of the study, normal saline was instilled into the femoral artery and the cardiorespiratory (CVR) responses were recorded. In the second part of the study, histamine in three different concentrations was instilled in different groups of rats and the CVR responses were recorded. In the third part of the study, 10 mM of histamine (optimal dose from concentration–response experiments) was instilled intra-arterially (i.a) after ipsilateral sciatic and femoral neurotomy (NX), bilateral vagotomy (VagX) and pre-treatment with lignocaine (+Ligno) in different groups of rats and the CVR responses were recorded.

## Results

**Figure 2:**
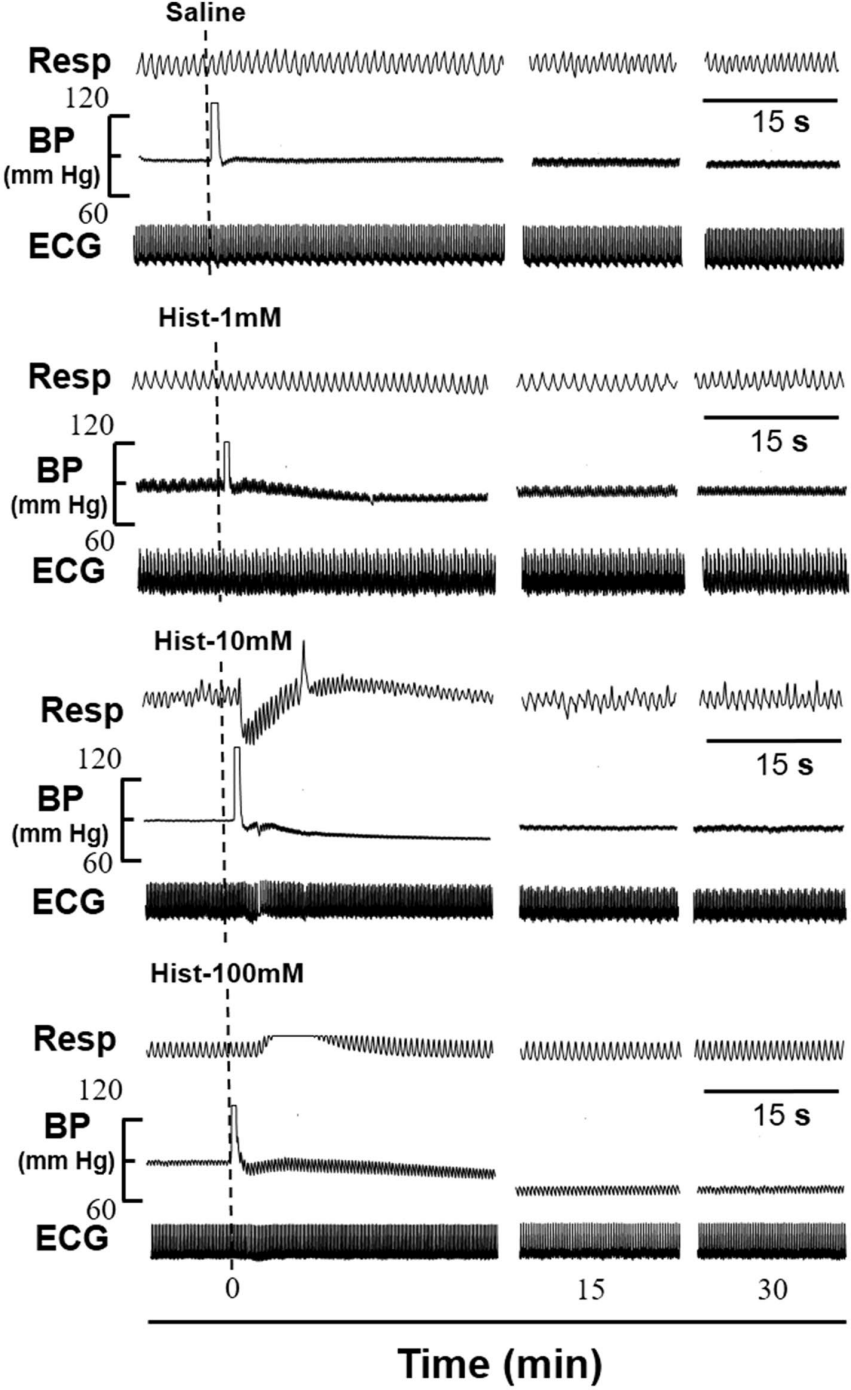
Representative prototypes of original recordings showing the effects of different concentrations of histamine (Hist) on respiration (Resp), blood pressure (BP) and electrocardiogram (ECG) as compared to normal saline control. The responses are shown at different time intervals as indicated in the lower panel. The points of instillation are shown by dotted lines. The horizontal line in each panel is 15 s for all parameters.

**Figure 3:**
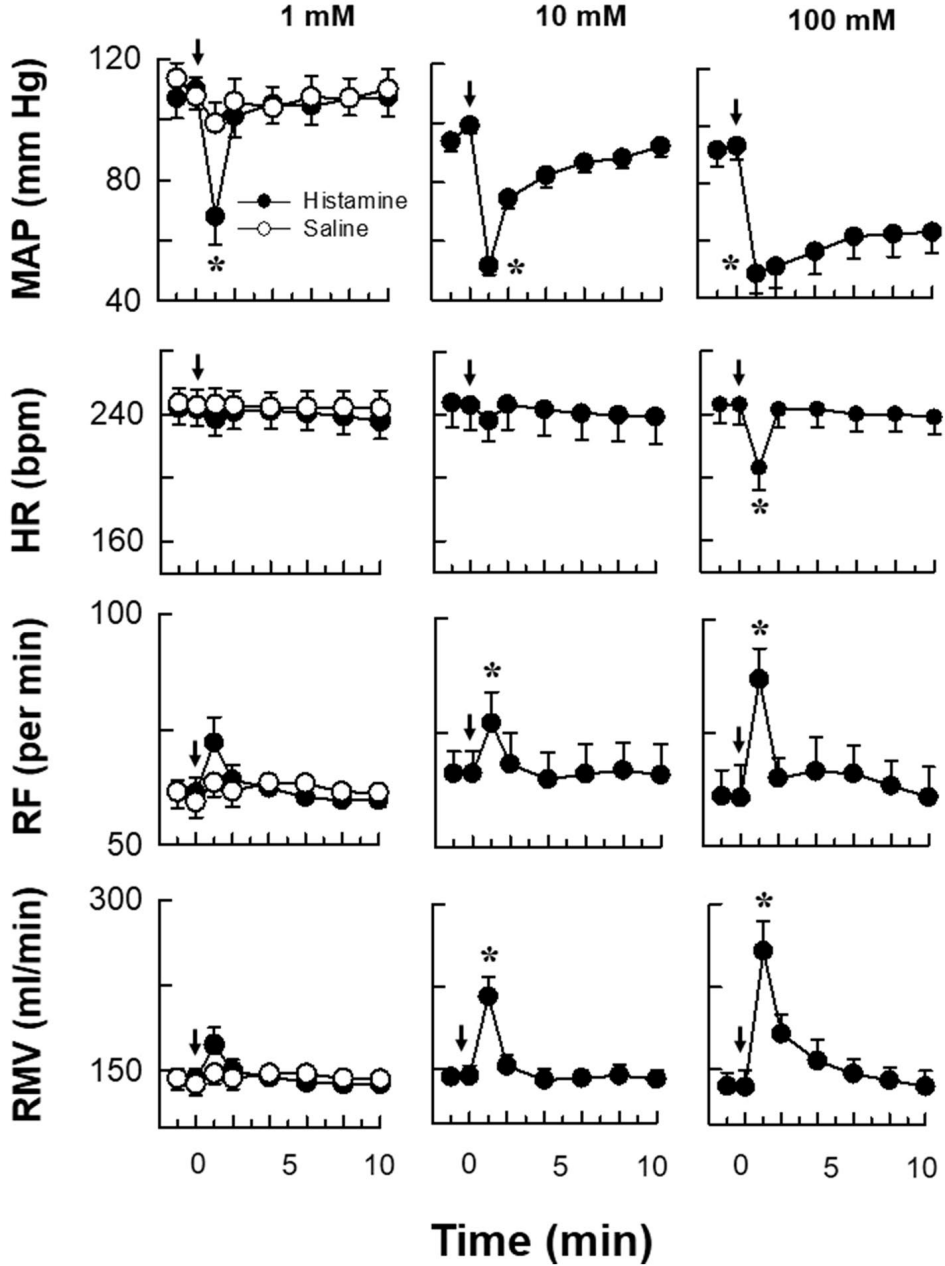
Time-response relationship of different histamine concentrations on mean arterial pressure (MAP), heart rate (HR), respiratory frequency (RF) and respiratory minute volume (RMV). The time matched control responses after saline are given in the left panel of each row along with 1 mM concentration of histamine. The values are in mean ± SEM from 6 experiments for each concentration. An asterisk (*) indicates significant difference of responses as compared with control group [*P* < 0.05, two sided Dunnett’s test] and with the initial value [*P* < 0.05, Paired t-test] for all concentrations of histamine. Arrows indicate the point of injection of saline/histamine.

**Figure 4:**
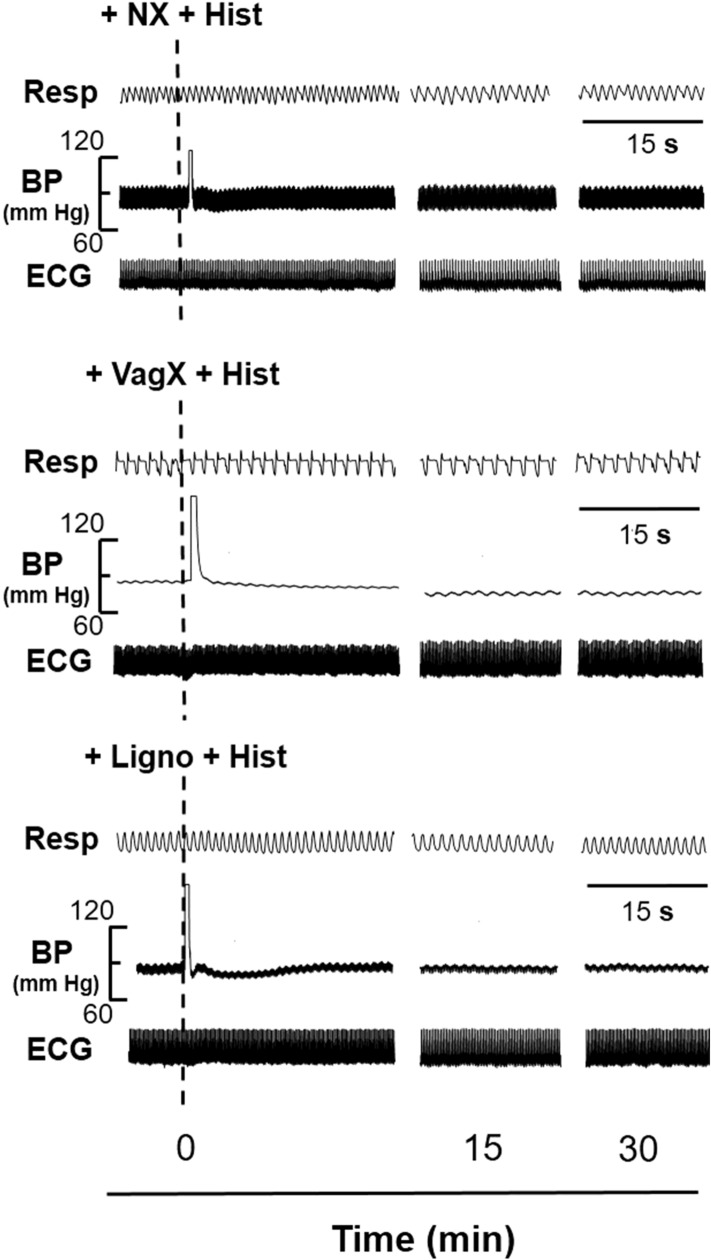
Representative prototypes of original recordings showing the effects of 10 mM histamine (Hist) on respiration (Resp), blood pressure (BP) and electrocardiogram (ECG), after ipsilateral sciatic and femoral neurotomy (+NX), bilateral vagotomy (+VagX) and pre-treatment with lignocaine (+Ligno). The responses are shown at different time intervals as indicated in the lower panel. The points of instillation of Hist after interventions are shown by dotted lines. The horizontal line in each panel is 15 s for all parameters.

**Figure 5:**
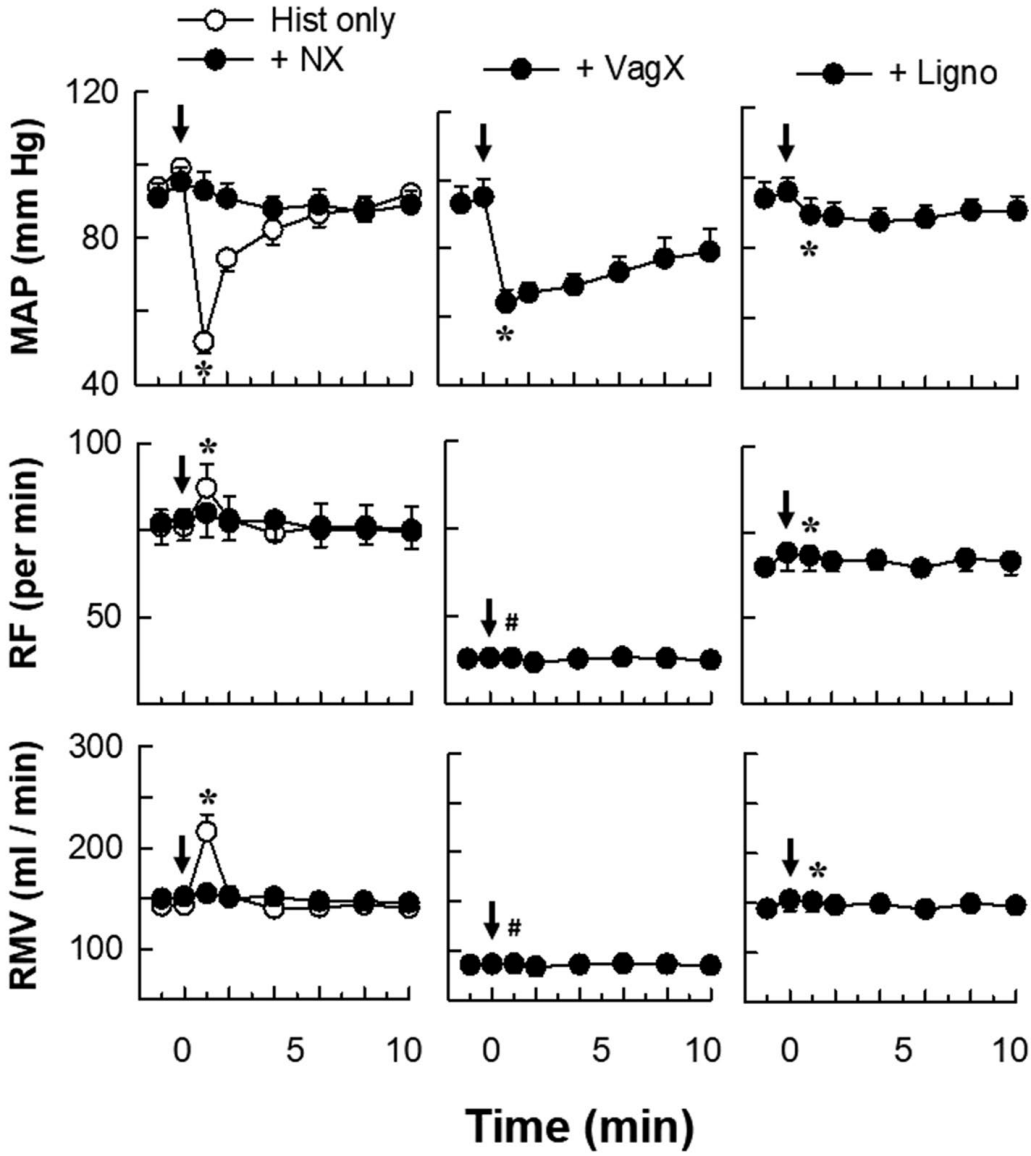
Time-response relationship of 10 mM histamine on mean arterial pressure (MAP), respiratory frequency (RF) and respiratory minute volume (RMV), after ipsilateral sciatic and femoral nerve sectioning (+NX), bilateral vagotomy (+VagX) and pre-treatment with lignocaine (+Ligno). The responses of histamine (10 mM) only group are given in the left panel of each row along with neurotomized group. The values are in mean ± SEM from 6 experiments in each group. (*) indicates *P* < 0.05, two sided Dunnett’s test as compared with the histamine (10 mM) only group; (#) indicates *P* < 0.05, Unpaired t-test as compared with the histamine (10 mM) only group. Arrows indicate the point of injection of histamine. HR responses are not included in this figure as 10 mM histamine did not produced significant HR alterations as compared to the control group [Fig. 3].

Histamine instillation in the femoral artery produced immediate hypotensive, bradycardiac, tachypnoeic and hyperventilatory responses of shorter latencies while equi-volume of saline (time matched control) did not produce any change in CVR responses for a period of 30 min [Figs. 2 and 3]. Saline was also instilled in each experiment 15 min prior to the instillation of histamine and no CVR alterations were observed. Table 1 gives the CVR parameters 10 s before and 1 min after the instillation of histamine/saline. The representative prototypes of the CVR changes are shown as original tracings [Figs. 2 and 4]. The transient phasic elevation of the BP upon histamine/saline administration represents the stimulus artefact as recorded by the BP transducer. Since significant changes in CVR parameters were observed within the first 10 min of histamine instillation, the CVR alterations during this interval are plotted as time-response relationships [Figs. 3 and 5].

**Table 1:** Cardiorespiratory (CVR) changes before and after instillation of saline/histamine.

	Saline/histamine	10 s before point of instillation	1 min after point of instillation	Alteration in CVR parameters (% of initial value)
MAP (mm Hg)	Saline	112.33 ± 6.91	97.50 ± 8.74	− 13.20
	Hist (1 mM)	110.00 ± 6.57	67.83 ± 9.22*	− 39.09
	Hist (10 mM)	99.17 ± 2.59	51.67 ± 3.19*	− 47.90
	Hist (100 mM)	93.00 ± 4.77	48.50 ± 6.69*	− 47.85
HR (beats per min)	Saline	254.50 ± 7.66	253.83 ± 7.88	− 0.26
	Hist (1 mM)	243.67 ± 11.23	237.33 ± 10.77	− 2.60
	Hist (10 mM)	245.83 ± 15.89	226.17 ± 13.08	− 8.00
	Hist (100 mM)	246.67 ± 12.60	206.33 ± 14.01*	− 16.52
RF (per min)	Saline	57.00 ± 6.87	63.83 ± 5.38	+ 12.00
	Hist (1 mM)	61.33 ± 3.13	72.17 ± 5.44	+ 17.67
	Hist (10 mM)	66.17 ± 4.78	77.17 ± 6.80*	+ 16.62
	Hist (100 mM)	60.67 ± 7.08	86.83 ± 6.75*	+ 43.12
RMV (ml/min)	Saline	149.95 ± 12.70	152.82 ± 11.31	+ 1.91
	Hist (1 mM)	151.28 ± 9.10	172.50 ± 15.51	+ 14.03
	Hist (10 mM)	143.17 ± 9.29	215.77 ± 17.56*	+ 50.71
	Hist (100 mM)	134.13 ± 15.19	257.45 ± 26.60*	+ 91.94

*MAP* mean arterial pressure, *HR* heart rate, *RF* respiratory frequency, *RMV* respiratory minute volume. In the last column, +/− indicates increase/decrease respectively. An asterisk (*) indicates significant alteration in CVR parameters as compared with the saline control group [*P* < 0.05, two sided Dunnett’s test] and with the initial value [*P* < 0.05, Paired t-test].

### Effects of different concentrations of histamine on CVR parameters

Instillation of histamine (i.a) elicited immediate hypotensive changes which were concentration dependent, minimal with 1 mM and maximal with 100 mM of concentration [Table 1; Figs. 2 and 3]. The decrease in MAP was significantly different than the time matched saline group [*P* < 0.05, two sided Dunnett’s test] or the corresponding initial value at all concentrations of histamine [*P* < 0.05, Paired t-test]. With 1 mM and 10 mM concentrations of histamine the MAP changes returned back to the initial value within 2–4 min of instillation while with 100 mM concentration, the MAP did not return back to the initial value and remained at the lower level for 30 min [Figs. 2 and 3]. Hence, the MAP response at 10 mM was taken as optimal response.

Bradycardiac responses were different significantly than the saline responses [*P* < 0.05, two sided Dunnett’s test] or from initial value [*P* < 0.05, Paired t-test], only with 100 mM concentration of histamine [Table 1; Fig. 3]. However, 1 mM and 10 mM of histamine did not produce significant bradycardiac changes. Histamine produced transient apnoea followed by hyperventilatory responses which were maximal at 100 mM concentration [Figs. 2 and 3]. The tachypnoeic and hyperventilatory responses with 1 mM concentration were not different from the saline group but the responses with 10 mM and 100 mM concentrations of histamine were different significantly from the saline control group [Table 1; Figs. 2 and 3; *P* < 0.05, two sided Dunnett’s test] and from the initial value [Table 1; Figs. 2 and 3; *P* < 0.05, Paired t-test].

The concentration response experiments show that at 1 mM concentration, the HR, RF and RMV responses were not significantly different than the saline responses. At 100 mM concentration the CVR responses were prolonged, indicating the possibility of mechanisms other than reflexes in producing these alterations. Therefore, it was inferred that 10 mM concentration of histamine produced optimal responses on all CVR parameters and hence, this concentration was chosen for the elicitation of reflex responses in the subsequent set of experiments. The latencies of the CVR responses with different concentrations of histamine are shown in Table 2.

**Table 2:** Mean ± SEM values of latencies of cardiorespiratory (CVR) responses after instillation of different concentrations of histamine.

Concentrations of histamine	Mean ± SEM latencies of CVR responses (s)		
	RF	MAP	HR
1 mM	3.17 ± 0.31	7.00 ± 0.37	4.50 ± 0.22
10 mM	2.33 ± 0.21	7.33 ± 0.33	3.67 ± 0.21
100 mM	4.17 ± 0.31	7.33 ± 0.42	3.17 ± 0.48

*RF* respiratory frequency, *MAP* mean arterial pressure; *HR* heart rate. The latencies of MAP responses are significantly different as compared with the RF and HR responses for all concentrations of histamine [*P* < 0.05, Unpaired t-test].

The mean ± SEM values of the water content (% of wet weight) of ipsilateral (histamine-instilled) paws were 76.39 ± 3.60, 78.08 ± 2.94 and 81.31 ± 1.05 with 1 mM, 10 mM and 100 mM concentrations of histamine respectively, while the water content of contralateral paws were 76.54 ± 3.61, 79.46 ± 2.68 and 81.53 ± 1.32 with 1 mM, 10 mM and 100 mM concentrations of histamine respectively. The water content in ipsilateral side was not different as compared to the contralateral side with all concentrations of histamine [*P* > 0.05, Unpaired t-test]. Likewise, the water content of ipsilateral paws in histamine groups (1 mM, 10 mM, 100 mM) were not significantly different [*P* > 0.05, Unpaired t-test] as compared with water content of ipsilateral paws in saline control group (75.32 ± 1.33). Further, the water contents of the ipsilateral and contralateral paws were not significantly different between groups treated with histamine at 1, 10 and 100 mM concentrations [*P* > 0.05, Unpaired t-test].

### Effects of somatic neurotomy, bilateral vagotomy and lignocaine pre-treatment on histamine-induced vasosensory reflex responses

Ipsilateral sciatic and femoral nerve sectioning (+NX) as such produced no alteration in the resting MAP, HR, RF and RMV up to 5 min. However, +NX attenuated the histamine-induced CVR changes significantly as compared with the histamine (10 mM) only group [Tables 1 and 3; Figs. 4 and 5; *P* < 0.05, two sided Dunnett’s test].

**Table 3:** Alteration in cardiorespiratory (CVR) parameters with 10 mM histamine after ipsilateral somatic neurotomy (+NX), bilateral vagotomy (+VagX) and pre-treatment with lignocaine (+Ligno).

CVR parameters	Alteration in CVR parameters (% of initial value)		
	+NX	+VagX	+Ligno
MAP	− 2.44	− 41.33	− 6.78
RF	+ 2.35	0.00	+ 1.20
RMV	+ 0.67	+ 0.49	+ 1.04

*MAP* mean arterial pressure, *RF* respiratory frequency, *RMV* Respiratory minute volume. + NX, + VagX and + Ligno attenuate MAP, RF and RMV parameters significantly [*P* < 0.05, two sided Dunnett’s test] as compared with the histamine (10 mM) only group [Table 1]. + / − indicates increase/decrease respectively.

Bilateral vagotomy (+VagX) per se produced immediate bradypnoea (RF from 60.67 ± 5.11 per min to 37.33 ± 0.42 per min) and hypoventilation (RMV from 133.07 ± 3.68 ml/min to 87.23 ± 7.45 ml/min) which remained at this level till the end of the experiments. Since the initial values of RF and RMV in vagotomized group were significantly different [Figs. 4 and 5; *P* < 0.05, Paired t-test] from that of the histamine (10 mM) only group, alterations in the CVR parameters were expressed as % of initial value and tested for significant difference. From Tables 1 and 3, it can be observed that + VagX significantly attenuated all the histamine-induced CVR changes [Figs. 4 and 5; *P* < 0.05, Paired t-test]. Bilateral vagotomy per se produced no change in the heart rate in our observations. This is most likely due to the low vagal tone in these animals.

Pre-treatment with lignocaine (+Ligno) did not alter the resting CVR parameters as such, but attenuated all the histamine-induced CVR changes significantly as compared with the histamine (10 mM) only group [Tables 1 and 3; Figs. 4 and 5; *P* < 0.05, two sided Dunnett’s test].

## Discussion

Instillation of histamine in a local segment of femoral artery evokes immediate hypotensive, bradycardiac, tachypnoeic and hyperventilatory responses, in contrast with equi-volume of saline in control experiments, eliminating the probability of ischemia/stretch-induced responses on the blood vessel wall. The responses with histamine are concentration dependent, maximal with 100 mM and minimal with 1 mM. The latencies are short (2–7 s) favouring the neuronal mechanisms involved in producing these responses. Ipsilateral neurotomy (+NX), pre-treatment with lignocaine (+Ligno) and bilateral vagotomy (+VagX) significantly attenuated histamine-induced reflex responses.

Perivascular nociceptive sensory nerve terminals have been implicated in the pain associated with diseases like migraine, angina pectoris, thrombo-embolism, claudication and myocardial infarction. Peripheral vascular disorders are involved in long-term cardiovascular alterations and other behavioural changes^4, 5^. Since, the peripheral blood vessels and large blood vessels of the heart are derived from the similar mesodermal origin therefore, it can be expected that peripheral blood vessels might be involved in sensing and signalling the perivascular environmental information to the CNS which in turn regulates the systemic cardiorespiratory parameters via vasosensory reflex responses.

The vasosensory reflex responses evoked by various agents like capsaicin, anandamide, αβ Me-ATP and bradykinin have already been demonstrated by the workers elsewhere^2–6^. Studies from our laboratory have demonstrated vasosensory reflex responses using *Mesobuthus tamulus* (BT) venom as a nociceptive tool^1, 7–9^. The BT venom is a combination of quite a lot of nociceptive agents like histamine, bradykinin, prostaglandins, serotonin etc.^12, 13^ which are also notable components of the inflammatory mediators. Hence, it becomes essential to identify the effect of individual components of the inflammatory mediators on the reflex responses. Recently, we have demonstrated the responses elicited after i.a. injection of bradykinin into a local segment of femoral artery^10^. Histamine is another pure nociceptive agonist and also a major constituent of the ‘inflammatory soup’. Hence, the present study was intended to assess the nature of vasosensory reflex responses elicited by histamine.

Earlier studies in dogs and cats have reported that injection of histamine into femoral vein causes a systemic fall in blood pressure as measured from femoral artery^14–16^. Further, administration of histamine into brachial vein has shown to produce veno-dilation and increased forearm blood flow in humans^17^. The above works by far, describe the effects of histamine only on BP, due to its presence in the systemic circulation. In the present study, histamine was instilled in a local segment of femoral artery and the changes in BP were studied along with changes in HR, RF and RMV. Further, the experimental design was modified using a 24G, double ported cannula, prepared in our laboratory^11^. This cannula helped us avoid the cannulation of carotid artery^2, 3, 6^, thereby minimizing the circulatory compromise to the CVR centres located in the pons and medulla. Retrograde cannulation of femoral artery made it an end artery through which minimal (100 µl) and constant volume of chemicals was injected. Thus, the instilled chemicals/saline remained in a local segment of femoral artery just proximal to the cannula and spillage into the systemic circulation was least possible. Absence of paw edema also supports the above statement.

The histamine-induced CVR alterations observed in this study, by and large were concentration dependent. 1 mM of histamine produced hypotensive response, but not bradycardiac, tachypnoeic and hyperventilatory responses, indicating the lower firing threshold for the afferents carrying impulses for MAP responses and higher threshold for the rest of the responses. It has been described elsewhere that the perivascular nociceptive afferents have characteristic thresholds and sensitivities that distinguish them from other sensory fibres. When these nociceptors are exposed to components of the ‘inflammatory soup’ such as histamine, bradykinin, serotonin and prostaglandins, various protein kinases get activated in the nociceptor terminals^18–22^. As a result the threshold of activation of these sensory afferents gets reduced due to phosphorylation of TRPV1 ion channels. This increases the excitability of the peripheral nerve terminal, producing a state of heightened sensitivity termed peripheral sensitization^23–26^. Further, 10 mM and 100 mM concentrations of histamine produced hypotensive, bradycardiac, tachypnoeic and hyperventilatory responses favouring the involvement of more than one afferents of different thresholds carrying the impulses. The delayed restoration of MAP and RMV responses at 100 mM concentration indicates the possibility of mechanisms, other than vasosensory reflexes in mediating these alterations and hence this concentration was avoided for further experiments. After analysing the histamine-induced response pattern, 10 mM concentration of histamine was chosen for the subsequent experiments.

In the neurotomized (+NX) group, histamine induced CVR responses are abolished, indicating that the primary afferents for these responses are running through the ipsilateral femoral and sciatic nerves. Similarly, the histamine-induced responses are significantly attenuated after lignocaine pre-treatment (+Ligno). Lignocaine, a local anesthetic agent causes membrane stabilisation by directly interacting with the voltage-gated Na+ channels of the perivascular afferent nerve terminals, thereby decreasing their excitability to chemical stimuli ^12^. Attenuation of histamine-induced CVR responses after +NX and +Ligno demonstrates that these responses are reflex in nature and originate from the local vascular bed. These findings also suggest that the afferents of the vasosensory reflex responses probably run as Aδ and C fibres through the ipsilateral femoral and sciatic nerves, travel along the lateral spino-thalamic system and give collateral projections to the medullary sympathetic and parasympathetic nuclei that mediate the reflex CVR alterations^12, 24, 26–28^.

Bilateral vagotomy (+VagX) per se produces bradypnoea and hypoventilation as the afferent and efferent respiratory fibres located in the vagi are transected. After i.a instillation of histamine in these animals, the hypotensive responses are slightly attenuated and the recovery from hypotension is delayed as compared with the histamine-only group. The possible explanation for this slow recovery of BP could be the abolition of afferent impulses from the compensating baroreceptor system that run through the vagi^29^. The RF and RMV responses to histamine are completely abolished after VagX which shows that the principal efferents for these responses are located in the vagi. However, the MAP responses are not blocked completely after VagX which clearly demonstrates the existence of additional efferent pathways mediating these responses, probably the sympathetic and parasympathetic fibres originating from the nucleus tractus solitarius and dorsal motor nucleus of vagus respectively^29^.

The role of histamine in the pathogenesis of inflammatory diseases involving peripheral blood vessels like Raynaud’s syndrome, polyarteritis nodosa, Kawasaki disease etc*.* is well known^24, 26^.Our study proposes an explanation for the CVR and autonomic alterations seen in such inflammatory diseases involving the medium-sized peripheral blood vessels.

In conclusion, presence of histamine in a segment of femoral artery evokes reflex CVR alterations of shorter latencies. The attenuation of reflex responses after ipsilateral femoral and sciatic neurotomy and pre-treatment with lignocaine shows that these responses are neuronal in nature, arising from the local vascular bed and most of their afferents are located in the ipsilateral somatic nerves. Further, attenuation of CVR responses in vagotomized rats indicates that the parasympathetic efferents for the vasosensory reflex responses are running through the vagus nerves. Our data supports the fact regarding the involvement of peripheral blood vessels in the reflex modulation of CVR system in health and diseases.

## Methods

The Animal Ethical Committee of the Institute of Medical Sciences, Banaras Hindu University, Varanasi, India approved the present study (Ref. No. Dean/2019/IAEC/1627 dated 17.11.2019). Animal experiments were performed in accordance with the US National Institute of Health “Guide for the Care and Use of Laboratory Animals (8^th^ edition)”. Study was carried out in compliance with the ARRIVE guidelines. Chemicals (histamine/saline/lignocaine) were instilled in the femoral artery retrogradely through a cannula and the volume was kept minimal (100 µl) and constant. Six animals were used in each group and the lab temperature was maintained at 28 ± 2 °C. Animals were sacrificed at the end of experiments by overdose of anesthesia. A schematic description of the experimental protocol is given in Fig. 1. The detailed methodology of dissection and recording was standardized, validated and published in our previous articles^10, 11^ and a brief relevant description is given below.

### Experimental animals and anesthesia

Male albino adult rats (Charles-Foster strain; 212.50 ± 10.50 g) were housed at 12:12-h dark/light cycle with provision of ad libitum food/water. Urethane (Merck, Germany) was freshly dissolved in distilled water (0.5 g/ml) and was injected to the rats intraperitoneally (1.5 g/kg). An additional dose (50–100 mg) of anesthesia was given, if required. After a satisfactory anesthesia, tracheostomy was performed by inserting a small polyethylene tube to keep the airway patent.

### Dissection and recording

The right femoral artery was cannulated retrogradely by a 24G, double ported polyethylene cannula filled with heparinized saline (20 IU/ml). The cannula facilitated instillation of chemicals/saline and blood pressure (BP) recording simultaneously, through its vertical and horizontal ports respectively. This cannula was in turn connected to a data acquisition system via a pressure transducer (Power Lab, AD Instruments, Australia). Further, needle electrodes connected in standard limb lead-II configuration provided the electrocardiographic (ECG) recordings on the computer screen. The respiratory movements were recorded using a force transducer. The mean arterial pressure (MAP), heart rate (HR) respiratory frequency (RF) and respiratory minute volume (RMV) were computed from the original recordings. At the end of experiments, both the hind paws were disarticulated at the level of ankle joint. The wet weights of paws were determined and were dried in an electric oven (90 °C) for a period of 72 h. The difference between wet weight and dry weight of a paw provided its water content and was expressed as percentage of wet weight. This methodology of paw edema estimation was standardized and described in detail in our previous publications^10, 11^.

### Histamine-induced CVR responses after ipsilateral somatic neurotomy

In this group of animals, before dissecting the femoral area, the animal was placed in left lateral position and the dorso-medial aspect of right thigh was dissected carefully using glass seekers, to expose the right sciatic nerve. Threads were passed around the sciatic nerve, so that it could be transected later, after femoral cannulation. Then, the animal was placed in supine position and dissection of right femoral triangle was performed to expose the femoral artery and nerve. The femoral artery was cannulated as mentioned earlier. Once the recording apparatus was set up, the right sciatic and femoral nerves were cut and the experimental protocol was followed as shown in Fig. 1.

### Histamine-induced CVR responses after bilateral vagotomy

In a separate group of animals, after tracheostomy, the carotid sheath was explored bilaterally using meticulous blunt dissection to isolate the carotid arteries and vagus nerves. Threads were passed under the vagi with minimal handling of the nerve. Then femoral cannulation was performed. The bilateral vagi were cut once the recording apparatus was ready and the experimental protocol was followed as shown in Fig. 1.

### Histamine-induced CVR responses after lignocaine pre-treatment

In this group of rats, after femoral cannulation and initial recording of CVR parameters, lignocaine (2%; 100 µl; i.a) was instilled and recording of CVR responses was performed for 5 min. This interval was found to be sufficient for lignocaine to exert its blocking effects on the perivascular afferents. From this 5 min CVR recording, we were also able to ascertain that i.a. lignocaine instillation as such did not cause any alteration in the baseline CVR parameters. This was followed by saline instillation and later by histamine, as shown in the experimental protocol [Fig. 1].

### Data analyses

The CVR responses were presented as mean ± SEM values. The statistical significance was analysed by comparing the data obtained from the MAP, HR, RF and RMV responses of histamine groups with the saline group. Similarly, the CVR responses to histamine in intervention groups were compared with the histamine (10 mM) only group. The comparisons between various groups were made by using paired/unpaired t-test and Post-Hoc correction using Dunnett’s test (two sided) for other observations using SPSS-16.0 software. *P* value of < 0.05 was considered significant.

### Chemicals and solutions

Histamine dihydrochloride salt was bought from Sigma-Aldrich Company, St. Louis, MO, USA and Heparin sodium (1000 U/ml) was procured from Biological Evans Ltd., Hyderabad, India. Stock solution of histamine (molar solution) was prepared in double distilled water (1.84 g/10 ml) and kept in refrigerator (2–8 °C). Further dilutions of histamine were made in 0.9% saline at the time of experiments. Lignocaine (2%; 21.3 mg/ml) was obtained from Cipla Pharmaceutical Company, Indore, India.

## Supplementary Information

Supplementary Information.
