# Supplementary material for: Ipsilateral somatic nerves mediate histamine-induced vasosensory reflex responses involving perivascular afferents in rat models
Source: Sci Rep. 2021 Jul 19;11:14648. doi: 10.1038/s41598-021-94110-x (PMC8290047; doi:10.1038/s41598-021-94110-x)
Supplement: Supplementary file 1 — Supplementary Information. [file 41598_2021_94110_MOESM1_ESM.pdf]

Ipsilateral somatic nerves mediate histamine-induced vasosensory reflex responses involving perivascular afferents in rat models

Ravindran Revand and Sanjeev K. Singh

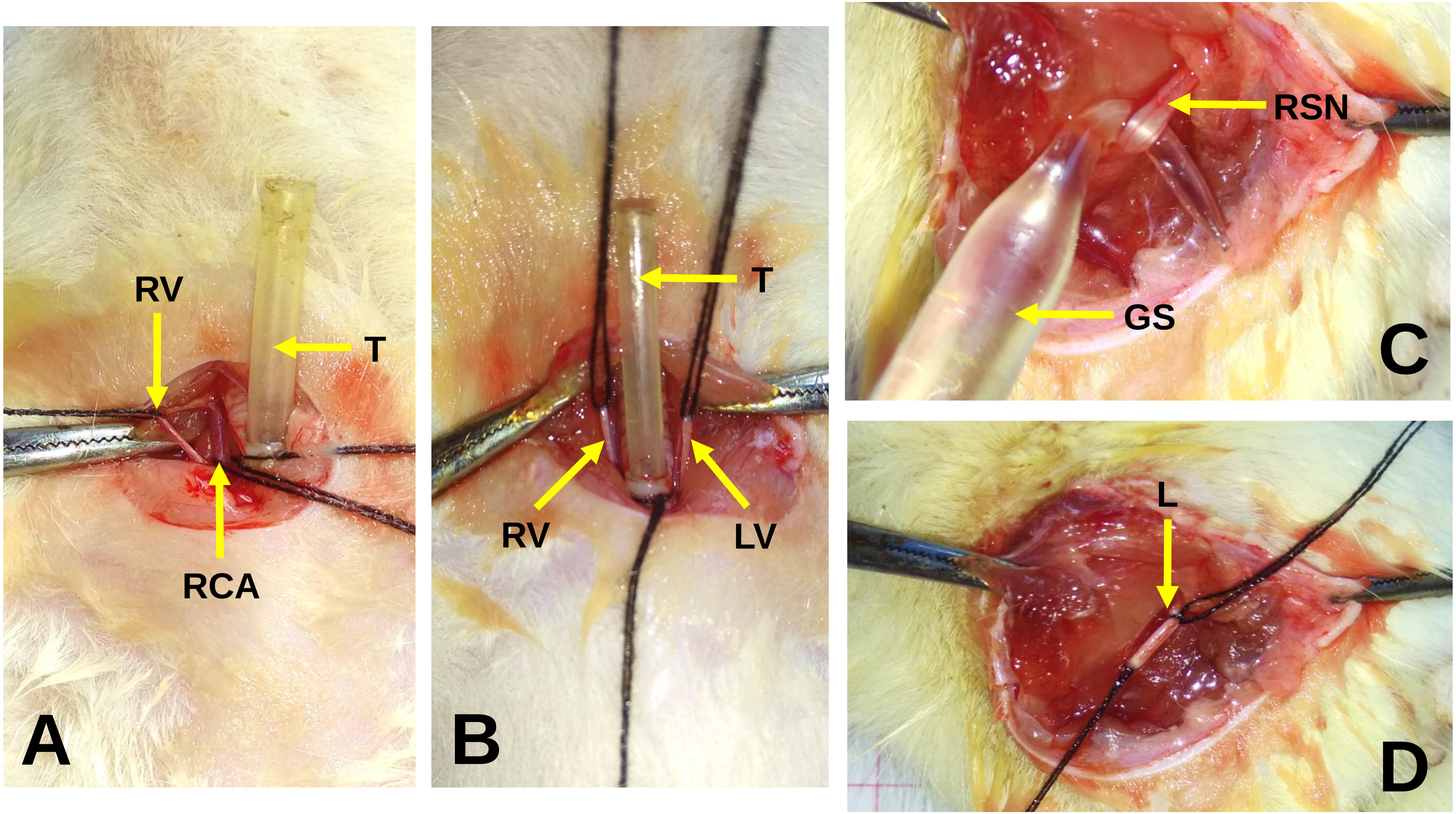

Figure S1: Photographic images showing the dissection procedure. **(A)** The right vagus (RV) nerve is dissected and isolated from the right carotid artery (RCA) using a thread. Tracheostomy (T) is performed to keep the airway patent. **(B)** The right vagus (RV) nerve and the left vagus (LV) nerves are separated from the surrounding structures using threads. **(C)** The right sciatic nerve (RSN) is dissected out using a glass seeker (GS). **(D)** Ligatures (L) are applied on the right sciatic nerve above and below the site of transection.
